# Downregulation of miR-342 is associated with tamoxifen resistant breast tumors

**DOI:** 10.1186/1476-4598-9-317

**Published:** 2010-12-20

**Authors:** Diana M Cittelly, Partha M Das, Nicole S Spoelstra, Susan M Edgerton, Jennifer K Richer, Ann D Thor, Frank E Jones

**Affiliations:** 1 Department of Pathology, University of Colorado Denver, School of Medicine, Aurora, Colorado, 80045, USA; 2 Department of Cell and Molecular Biology, Tulane University, New Orleans, Louisiana, 70118, USA

## Abstract

**Background:**

Tumor resistance to the selective estrogen receptor modulator tamoxifen remains a serious clinical problem especially in patients with tumors that also overexpress HER2. We have recently demonstrated that the clinically important isoform of HER2, HERΔ16, promotes therapeutically refractory breast cancer including resistance to endocrine therapy. Likewise additional breast tumor cell models of tamoxifen resistance have been developed that do not involve HER2 overexpression. However, a unifying molecular mechanism of tamoxifen resistance has remained elusive.

**Results:**

Here we analyzed multiple cell models of tamoxifen resistance derived from MCF-7 cells to examine the influence of microRNAs (miRNAs) on tamoxifen resistance. We compared miRNA expression profiles of tamoxifen sensitive MCF-7 cells and tamoxifen resistant MCF-7/HER2Δ16 cells. We observed significant and dramatic downregulation of miR-342 in the MCF-7/HER2Δ16 cell line as well as the HER2 negative but tamoxifen resistant MCF-7 variants TAMR1 and LCC2. Restoring miR-342 expression in the MCF-7/HER2Δ16 and TAMR1 cell lines sensitized these cells to tamoxifen-induced apoptosis with a dramatic reduction in cell growth. Expression of miR-342 was also reduced in a panel of tamoxifen refractory human breast tumors, underscoring the potential clinical importance of miR-342 downregulation. Towards the goal of identifying direct and indirect targets of miR-342 we restored miR-342 expression in MCF-7/HER2Δ16 cells and analyzed changes in global gene expression by microarray. The impact of miR-342 on gene expression in MCF-7/HER2Δ16 cells was not limited to miR-342 *in silica *predicted targets. Ingenuity Pathways Analysis of the dataset revealed a significant influence of miR-342 on multiple tumor cell cycle regulators.

**Conclusions:**

Our findings suggest that miR-342 regulates tamoxifen response in breast tumor cell lines and our clinical data indicates a trend towards reduced miR-342 expression and tamoxifen resistance. In addition, our results suggest that miR-342 regulates expression of genes involved in tamoxifen mediated tumor cell apoptosis and cell cycle progression. Restoring miR-342 expression may represent a novel therapeutic approach to sensitizing and suppressing the growth of tamoxifen refractory breast tumors.

## Background

Nearly, 70% of breast cancer patients develop tumors expressing the estrogen receptor (ERα) and are candidates for endocrine therapy. The selective ERα modulator tamoxifen, is the most commonly prescribed endocrine therapy, but 30-40% of patients fail adjuvant tamoxifen therapy and nearly all patients with metastatic disease develop tamoxifen resistance [1]. Unfortunately, *de novo *and acquired tumor resistance to tamoxifen therapy remains a poorly understood and serious clinical problem.

Multiple causal events have been associated with anti-estrogen resistance including loss of ERα expression [2], selection of ERα mutants [3,4], and cross-talk between the type I receptor tyrosine kinase family resulting in ligand-independent activation of the ERα [5]. Several clinical studies implicate the HER2 receptor tyrosine kinase as a significant risk for tamoxifen failure. Approximately half of the ERα positive tumors also express HER2 and over 70% of these patients may exhibit *de novo *tamoxifen resistance [6,7]. A large percentage of HER2/ERα positive tumors acquire estrogen-independence and therefore continue to grow when patients are estrogen depleted [6].

MicroRNAs (miRNAs) are short (~22 bp), single-stranded, non-coding RNAs that suppress gene expression by binding the 3' UTR of target gene mRNAs. They are thought to regulate up to one-third of the human genome by targeting mRNAs for cleavage or translational repression and miRNAs have recently been identified as key players in cellular processes including self-renewal, differentiation, growth and apoptosis, all of which are deregulated in carcinogenesis. Several miRNAs have been shown to be differentially regulated in breast cancer [8], and individual miRNAs that contribute to tumorigenicity, invasion and metastasis have been identified [9]. Recently, breast cancer prognostic markers such as ERα and HER2 have been shown to be regulated by miR-221/222 and miR-125, respectively [10,11]. Importantly, miR-221/222 and the BCL-2 targeting miR-15a/16 have been shown to contribute to tamoxifen resistance [11-13], implicating multiple miRNAs as important modulators of tamoxifen response. As multiple mechanisms contribute to the acquisition of tamoxifen resistance, it is likely that additional miRNA regulators of endocrine response remain to be identified.

Recently we demonstrated that the oncogenic splice isoform of HER2, HER2Δ16, is associated with metastatic breast cancer and resistance to both trastuzumab [14] and endocrine therapy [12,15]. Here we show that deregulation of miR-342 contributes to tamoxifen resistance in multiple models of tamoxifen resistance including the HER2Δ16 overexpression model. Specifically, we demonstrate that miR-342 is downregulated in tamoxifen resistant breast tumor cell lines and tamoxifen refractory human breast tumors. We propose that miR-342 may emerge as an important marker for tamoxifen response as well as a potential therapeutic.

## Methods

### Cell Lines

Human mammary carcinoma cell line MCF-7 was purchased from the American Type Culture Collection (Manassas, VA) and maintained in MEM supplemented with 10% fetal bovine serum (FBS). The tamoxifen sensitive MCF-7/HER2, MCF-7/pcDNA, and the tamoxifen-resistant MCF-7/HER2Δ16 cell lines have been described elsewhere [12,14,15]. The tamoxifen resistant MCF-7 variants TAMR1 and LCC2 have been described previously [16,17]. To generate the miR-342 expressing MCF-7/HER2Δ16 cell line a 342 bp sequence containing the pre-miR-342 sequence (GRCh37:14:100575892:100576190:1) flanked by 100 bp upstream and downstream was prepared as a minigene (Integrated DNA Technologies) and subcloned into pCMV-puro-silencer (Ambion). The same vector expressing a short scrambled sequence (pCMV-puro-NC) was used as a control. Either pCMV-miR-342 or pCMV-puro-NC were transfected into MCF-7/HER2Δ16 cells using Fugene6 (Roche) and stable clones were isolated.

### miRNA Expression Profiling

Total RNA was isolated using miRVANA RNA Isolation System (Ambion) and integrity of samples was confirmed using a Bioanalyzer (Agilent). For miRNA profiling, biological duplicates of cells cultured for 48 h in CS-FBS and then treated with either 100 pM 17-β-estradiol alone or in combination with 1.0 μM 4-hydroxytamoxifen (TAM) were analyzed. Microarray assay was performed and analyzed using a service provider (LC Sciences) using LC-Science microRNA arrays miRHuman_11.0 which detects miRNA transcripts listed in Sanger miRBase Release 11.0.

### Northern Blot Analysis

Total RNA was isolated using miRVANA RNA isolation Kit (Ambion) and 10 μg of RNA was separated by electrophoresis on a 15% TBE/urea gel. RNA was transferred to a Hybond NX membrane (Amersham/Pharmacia), UV-crosslinked, and incubated in pre-hybridization solution (6X SSC, 5X Denhart's solution, 0.2% SDS) for 1 h at 30°C. Membranes were then hybridized (42°C for 16 h) with P^32^-labeled DNA probes corresponding to the complementary sequences of the mature miR-342-3p. The blots were exposed for 48 to 72 hr and developed using Molecular Dynamics Phosphoimaging.

### Quantification of miR-342

Expression of miR-342 was quantitated by qRT-PCR from total RNA exactly as described elsewhere [12].

### Analysis of EVL mRNA by Quantitative Reverse Transcription PCR

Expression of EVL was quantitated by qRT-PCR from total RNA exactly as described elsewhere [12] using the oligonucleotide primers 5'-TGCTGCTCCATCACTTGTCT and 5'-CTCCAATGCAATGCTGTTTG.

### Breast Tumor Samples

The patient population used for this study has been described in detail elsewhere [18]. Formalin fixed paraffin-embedded human primary breast tumors were obtained from the Department of Pathology at the University of Colorado. Tumor samples were collected from 1978 to 1993 and patients underwent tamoxifen therapy. Additional immunohistochemical, clinical, and pathological details of this cohort have been described elsewhere [18-21].

### *In situ *hybridization of miR-342

*In situ *hybridization (ISH) of 6 μm sections from archived FFPE primary breast tumor specimens was performed using standard techniques and double-DIG Locked Nucleic Acid-modified DNA probe complementary to mature hsa-miR-342-3p or scrambled control (Exiqon) according to the manufacturer's instructions. Each section was scored by comparing staining intensity of stably expressing miR-342 MCF-7/HER2Δ16 cells given a score of 3 and the MCF-7/HER2Δ16 cells with a score of 0 to the staining of tumor cells present in each section. Two independent observers were blind to sample identification and independently scored each slide. Each sample was given a final score based upon consensus.

### 3-(4,5-dimethylthiazol-2-yl)-2,5-diphenyltetrazolium Bromide Assay

Cell proliferation was measured as a function of metabolism by 3-(4,5-dimethylthiazol-2-yl])-2,5-diphenyltetrazolium bromide (MTT; Sigma, St. Louis, MO) assay exactly as described elsewhere [22] with detailed modifications [12]. Each sample was prepared in triplicate and the data represent the mean and SE of at least three independent experiments. Data was normalized to control-mock treated cells. Statistically significant differences between data sets were determined using paired Student's t test.

### Apoptosis Assay

Cell death as a result of apoptosis was quantitated by measuring mono- and oligonucleosomes release using the Cell Death Detection ELISA PLUS Kit (Roche) following the manufacturer's instructions.

### Inhibition of miR-342

Each cell line plated at 3000 cells per well in a 96-well tissue culture plate was cultured for 24 hrs in CS-MEM and then transfected with 50 nM of miRIDIAN miRNA inhibitor non-specific control 1 or miRIDIAN miRNA inhibitor hsa-miR-342-3p (Dharmacon) using Hyperfect Reagent (Qiagen) according to the manufacturer's instructions. At one day post-transfection cells were treated with 100 pM 17-β-estradiol alone or in combination with 1.0 μM 4-hydroxytamoxifen and a MTT growth assay was performed at five days post-transfection or cell death as a result of apoptosis was quantitated by measuring mono- and oligonucleosomes release using the Cell Death Detection ELISA PLUS Kit (Roche) following the manufacturer's instructions. Each sample was prepared in triplicate and the data represent the mean and SE of at least three independent experiments. Statistically significant differences between data sets were determined using paired Student's t test.

### Transient Expression of miR-342

Pre-miR miRNA Precursor Molecules (Ambion) for hsa-miR-342-3p were transfected into cell lines at the indicated concentrations using Hyperfect (Qiagen).

### 3' UTR Reporter Assay

MCF-7 cells were plated at 5000 cells per well in a 48 well plate and transfected with 20 nM of hsa-miR-342-3p (Ambion) or pre-miR negative control (Ambion) using Hyperfect. The next day cells were transfected with 1.0 μg of with pMir target firefly luciferase reporter plasmid containing 3' UTR sequences from BMP7 or GEMIN4 (Origene) and 1.0 μg of renilla luciferase expression plasmid pRL-SV40 with Fugene 6. At 48 hrs post-transfection cells were analyzed using the Dual Luciferase Assay Kit (Promega) according to the manufacturer's instructions. Each sample was prepared in duplicate and the entire experiment was repeated three times.

### Microarray Gene Expression Analysis

Total RNA from biological triplicates of MCF-7/HER2Δ16-miR-342 and MCF-7/HER2Δ16-puro-NC was labeled and hybridized to Affymetrix GeneChip Human Exon 1.0 ST Array and data was analyzed using GeneSpring GX 9.0 (Agilent) software at the University of Colorado Denver DNA Microarray Core Facility. Molecular pathway and connectively maps of the dataset was performed using Ingenuity Pathway Analysis software.

## Results

### miR-342 Expression is Suppressed in Tamoxifen Resistant Breast Tumor Cells

We have recently shown that ectopic expression of HER2Δ16, but not wild-type HER2 promotes tamoxifen resistance and estrogen independence of ERα positive MCF-7 cells in part through modulation of miR-15a/16 [12,15]. To identify additional miRNAs that influence tamoxifen resistance we compared global miRNA expression profiles of tamoxifen sensitive MCF-7/pcDNA cells to tamoxifen resistant MCF-7/HER2Δ16 cells after treatment with 100 pM of 17-β-estradiol (E2) alone or in combination with 1.0 μM 4-hydroxytamoxifen (TAM). We first examined the impact of TAM on miRNA expression in the two cell lines. We reasoned that miRNAs associated with acquired TAM resistance may be altered during TAM treatment. However no miRNAs were significantly (*p *< 0.05) altered during TAM treatment of MCF-7/pcDNA cells and only expression of miR-125a was significantly (*p *< 0.05), albeit modestly (1.2 fold increase), altered during TAM treatment of MCF-7/HER2Δ16 cells. Results from this one set of cell lines suggest that TAM treatment fails to significantly alter miRNA expression.

**Figure 1:**
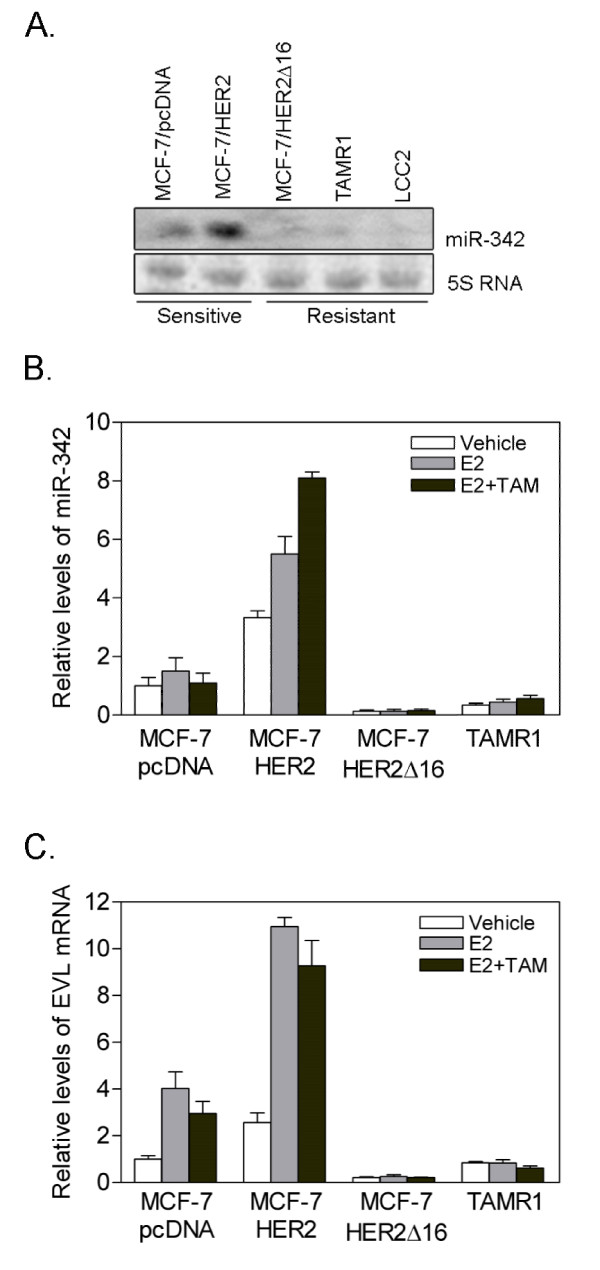
**Expression of miR-342 is suppressed in tamoxifen resistant breast tumor cells**. (A) Total RNA was isolated from tamoxifen-sensitive (MCF-7/HER2, MCF-7/pcDNA) and tamoxifen-resistant (MCF-7/HER2Δ16, TAMR1) cell lines cultured for 48 hr in 5% CS-FBS MEM and treated for 24 hr with 100 pM 17-β-estradiol (E2) and 1 μM 4-hydroxytamoxifen (TAM). RNA was analyzed by Northern Blot using DNA probes specific for miR-342-3p (miR-342) and the 5S RNA loading control. (B,C) Total RNA was isolated from each cell line treated with vehicle or E2 alone or in combination with TAM for 24 hr and (B) miR-342 or (C) EVL expression was analyzed by qRT-PCR and normalized to actin mRNA values. Levels are relative to vehicle-treated MCF-7/pcDNA cells. Data represents the mean +/- SE of triplicate samples.

We next attempted to identify potential miRNA modulators of TAM response by comparing miRNA expression profiles between MCF-7/pcDNA and MCF-7/HER2Δ16 cells while both cell lines are undergoing acute tamoxifen treatment. Using this criteria we identified five significantly (*p *< 0.05) altered miRNAs (Table 1). We focused follow-up analysis on miR-342-3p (miR-342) which was the most dramatically altered miRNA in the tamoxifen resistant MCF-7/HER2Δ16 cells and miR-342 has recently been shown to be associated with ERα(+) and HER2 (+) breast tumors [23]. We first determined if loss of miR-342 was a common feature of tamoxifen resistance by comparing miR-342 expression by northern blot in multiple tamoxifen sensitive and resistant MCF-7 variants. Interestingly, the two tamoxifen sensitive MCF-7/pcDNA and MCF-7/HER2 cell lines [12,15] expressed high levels of miR-342 whereas the tamoxifen resistant MCF-7/HER2Δ16 [12,15], TAMR1 [17], and LCC2 [16] cell lines all exhibited dramatically suppressed levels of miR-342 (Figure 1A). Consistent with these results, qRT-PCR analyses showed that miR-342 is suppressed in the tamoxifen resistant MCF-7/HER2Δ16 and TAMR1 cell lines when compared to tamoxifen sensitive MCF-7/pcDNA cells (Figure 1B). With the exception of the tamoxifen sensitive MCF-7/HER2 cells, treatment with E2 alone or in combination with TAM did not significantly alter miR-342 expression (Figure 1B). Importantly, expression of miR-342-5p was between 10-80 fold lower than miR-342-3p and in many cases miR-342-5p expression was at the limits of qRT-PCR sensitivity (Additional File 1).

**Table 1:** MicroRNAs Significantly Altered in Tamoxifen Treated MCF-7/pcDNA vs. MCF-7/HER2Δ16 Cells

miRNA	MCF-7/pcDNA		MCF-7/HER2Δ16		Fold Change E+TAM HER2Δ16/pcDNA	*p*
	**E**	**E+TAM**	**E**	**E+TAM**		

miR-1308	459	514	2434	1772	3.45	0.033

miR-125a-5p	5090	6432	5909	7179	1.12	0.034

miR-23a	7798	7427	6473	5191	-1.43	0.013

miR-1180	815	797	189	188	-4.24	0.036

miR-342-3p	1960	1386	166	166	-8.35	0.031

Because intronic miRNA expression is commonly coordinated with expression of the miRNA host-gene we also examined expression of the miR-342 host-gene Ena/Vasp-like (EVL) in the tamoxifen sensitive and resistant cell lines. In each cell line we observed a concordance between the levels of EVL expression and miR-342 expression (Figure 1C) suggesting that miR-342 expression is directly coupled to expression of EVL. Similar coordinate expression between EVL and miR-342 has been reported in colorectal cancer [24]. In breast cancer independent studies have shown that similar to EVL, expression of miR-342 is associated with ERα(+) breast tumors http://www.oncomine.org[23,25-27] further supporting co-expression of miR-342 with EVL in ERα(+) tumors.

### miR-342 Expression is Suppressed in Primary Breast Tumors Associated with Tamoxifen Failure

**Figure 2:**
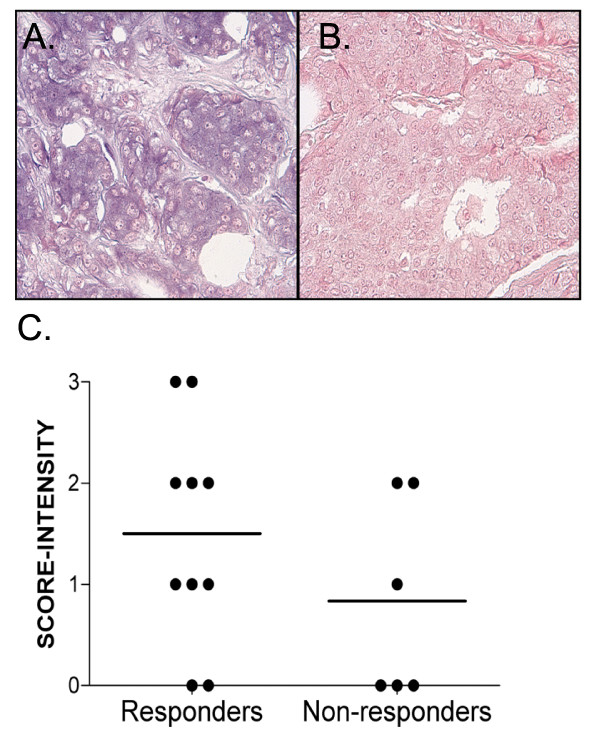
**Expression of miR-342 is suppressed in primary breast tumors associated with tamoxifen failure**. (A,B) Photomicrograph of primary breast tumors stained for miR-342 expression by ISH from patients that (A) responded or (B) failed tamoxifen therapy. (C) Scatter plot of primary breast tumor miR-342 ISH scores (range 0-3 with 3 highest staining levels) from 10 responders and 6 non-responders during tamoxifen treatment.

To determine if miR-342 downregulation was associated with clinical tamoxifen failure, we used DIG-labeled LNA modified miRNA probes to detect miR-342 in primary human breast tumors by ISH and independently scored each tumor on a scale of 0-3 with 3 being the most intense miR-342 staining (Additional File 2 and Figure 2A, B). We examined 16 primary breast tumors ER(+) from patients who underwent tamoxifen treatment, 6 developed recurrences and metastasis during tamoxifen treatment (non-responders), and 10 had non-recurrent disease (responders). Although we failed to obtain significance due to the small number of tumor samples, we did find that responders had nearly two-fold the levels of miR-342 expression when compared to tumors from patients that failed tamoxifen therapy (1.5 +/- 0.3 vs. 0.8 +/- 0.4)(Figure 2C).

### Suppression of miR-342 Expression Promotes Tamoxifen Resistance

**Figure 3:**
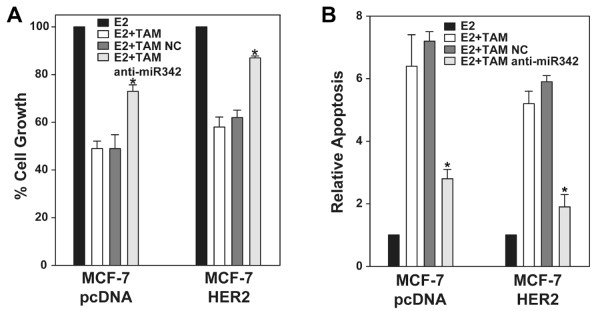
**Suppression of miR-342 promotes tamoxifen resistance**. The tamoxifen sensitive MCF-7/pcDNA and MCF-7/HER2 cell lines cultured for 48 hr in 5% CS-FBS MEM were transfected with anti-miR-342 or anti-miR negative control (NC) and the next day treated with 100 pM E2 alone or in combination with 1.0 μM TAM for five days. (A) MTT assay was used to quantitate cell growth and (B) apoptosis was quantitated using a Cell Death Detection ELISA. Data is represented as mean +/- SE of three independent experiments relative E2 treated MCF-7/pcDNA cells. Asterisks indicate samples with significant differences as determined by paired Student's *t *test (A, *p *< 0.01; B, *p *< 0.005).

To determine if modulation of miR-342 expression impacts tamoxifen response we first suppressed miR-342 expression in the tamoxifen sensitive MCF-7/pcDNA and MCF-7/HER2 cell lines. Consistent with our previously published results [12] treatment of MCF-7/pcDNA and MCF-7/HER2 cells with 1.0 μM of tamoxifen resulted in growth suppression with an increase in cellular apoptosis (Figure 3). Transient transfection of each cell line with a miR-342 inhibitor promoted partial but significant tamoxifen resistance (*p *< 0.01) with a significant decrease in tamoxifen induced apoptosis (*p *< 0.005)(Figure 3). These results suggest that expression of miR-342 sensitizes breast tumor cells to tamoxifen treatment and tamoxifen resistance can be acquired through suppression of miR-342.

### Restored miR-342 Expression Sensitizes Resistant Breast Tumor Cells to Tamoxifen

**Figure 4:**
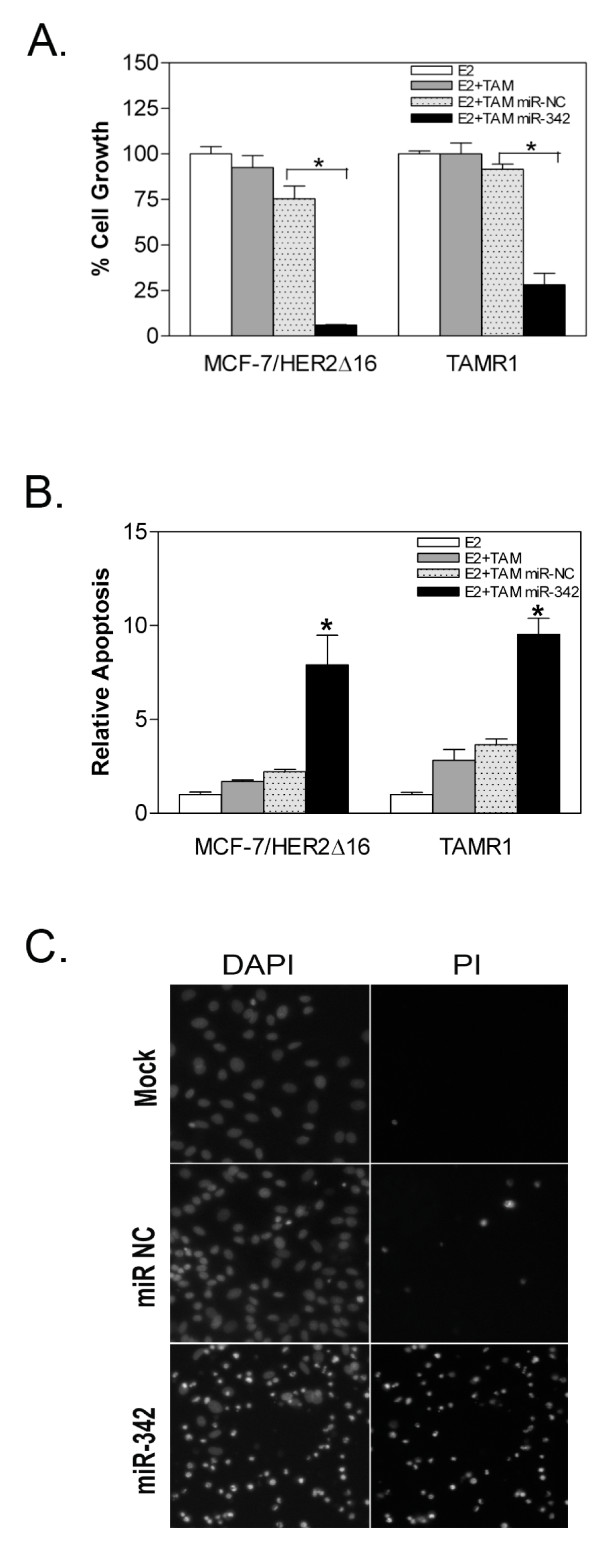
**Restored miR-342 expression sensitizes resistant cells to tamoxifen**. (A-C) Tamoxifen resistant cell lines were cultured for 24 hr in 5% CS-FBS MEM then transfected with transfection reagent alone, 20 nM of scrambled precursor negative control (miR-NC), or 20 nM of miR-342-3p precursor (miR-342). At 24 hr post-transfection cells were treated for 96 hr with 100 pM of E2 alone or in combination with 1.0 μM TAM. (A) MTT assay was used to measure proliferation as a function of metabolism, (B) apoptosis was assayed by cell death ELISA, or (C) cells were stained with DAPI and propidium iodide (PI). (A,B) Each experiment was repeated three times and the data is represented as the mean +/- SE of each cell line relative to the E2 treated sample. Asterisks indicates significant changes (*p *< 0.001) relative to E2 treated samples.

To determine if miR-342 expression sensitizes breast tumor cells to tamoxifen therapy, we reintroduced miR-342 expression into the tamoxifen resistant MCF-7/HER2Δ16 and TAMR1 cell lines. Restoration of miR-342 expression in tamoxifen-resistant MCF-7/HER2Δ16 and TAMR1 cells sensitized both cell lines to tamoxifen treatment with a significant reduction in cell growth (Figure 4A) and significant increase in tamoxifen-induced apoptosis (Figure 4B). Similar results were obtained when MCF-7/HER2Δ16 cells were stained with DAPI and Propidium Iodine (PI) 48 hr after tamoxifen treatment. A dramatic increase in the number of PI positive (dead cells) was observed in response to tamoxifen treatment but only in cells where miR-342 expression was restored (Figure 4C). Taken together these results implicate miR-342 expression as an important mediator of tamoxifen induced apoptosis in breast tumor cells.

### miR-342 Regulates Genes Associated with Tumor Cell Death and Cancer Pathways

**Figure 5:**
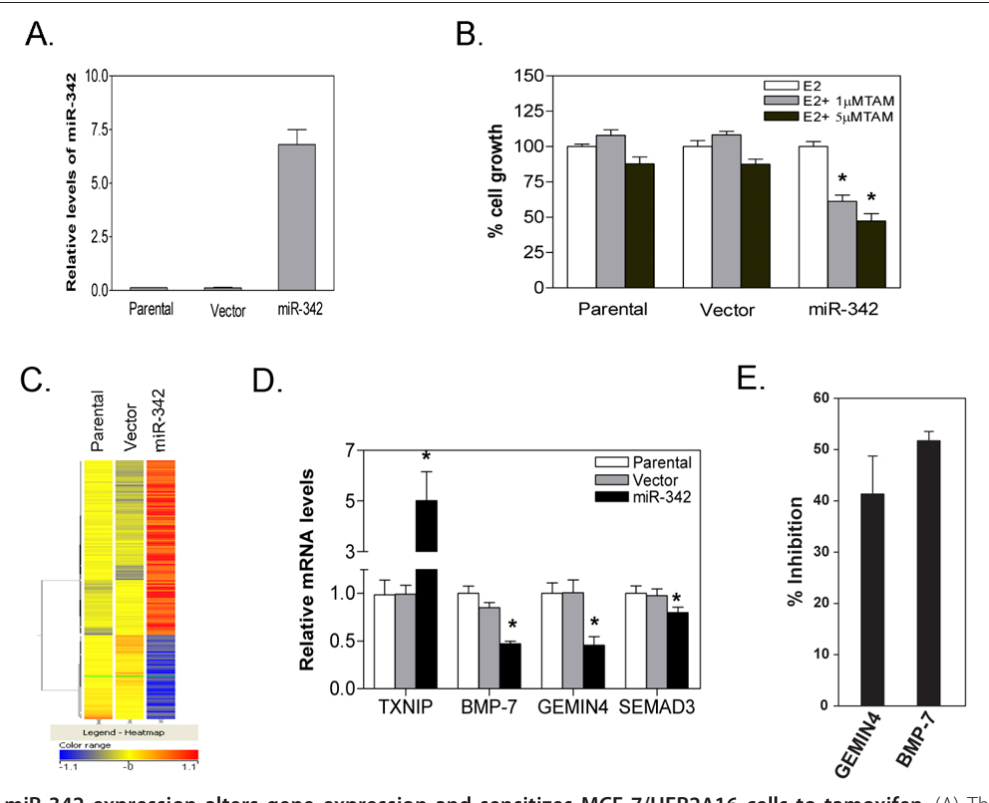
**Stable miR-342 expression alters gene expression and sensitizes MCF-7/HER2Δ16 cells to tamoxifen**. (A) Three independent total RNA samples purified from MCF-7/HER2Δ16 (Parental), stable MCF-7/HER2Δ16 cell lines expressing pCMV-puro-NC (Vector) or pCMV-miR-342 (miR-342) cultured for 48 hr in CS-FBS MEM were analyzed for miR-342 expression by qRT-PCR. (B) Each indicated cell line was cultured for 24 hr in 5% CS-FBS MEM and treated for 96 hr with 100 pM of E2 alone or in combination with 1.0 μM or 5 μM TAM. Each MTT experiment was repeated three times and the data is represented as the mean +/- SE relative to the E2 treated sample. Asterisks indicates significant changes (*p *< 0.04). (C) Heat map of microarray expression analysis of genes significantly altered (*p *< 0.001) by at least 1.5 fold in stable miR-342 expressing MCF-7/HER2Δ16 cells. (D) Microarray validation by qRT-PCR of three suppressed miR-342 direct target genes (SEMAD, BMP7, GEMIN4) and an upregulated indirect miR-342 target gene (TXNIP), normalized to β-actin, and expressed relative to parental MCF-7/HER2Δ16 cells. Asterisks indicate significant differences (*p *< 0.05). (E) MiR-342 inhibition of GEMIN4 and BMP7 3' UTRs. MCF-7 cells were transfected with 20 nM of hsa-miR-342-3p (Ambion) or pre-miR negative control (Ambion) followed by pMir target firefly luciferase reporter plasmid containing 3' UTR sequences from BMP7 or GEMIN4 (Origene) and a renilla luciferase expression vector. At 48 hrs post-transfection cells were analyzed using the Dual Luciferase Assay Kit (Promega) according to the manufacturer's instructions. Each sample was prepared in duplicate and the entire experiment was repeated three times. Data represents mean +/- SE percent inhibition of luciferase activity of miR-342 transfected cells relative to pre-miR negative control.

Despite the efforts of several laboratories, the physiological targets for miR-342 remain uncharacterized. Bioinformatic analyses predict that miR-342 can target between 169 to 1069 different genes. In order to identify potential direct and indirect targets of miR-342 we stably restored miR-342 levels in the tamoxifen resistant MCF-7/HER2Δ16 cell line and performed a gene expression analysis comparing vector control to miR-342 expressing MCF-7/HER2Δ16 cell clones. MCF-7/HER2Δ16 cells stably transfected with an expression vector containing the hsa-miR-342-3p precursor sequence (miR-342), resulted in an approximate 7-fold increase in miR-342 levels when compared to the parental MCF-7/HER2Δ16 cell line (Figure 4A) with undetectable levels of miR-342-5p expression (Additional File 3). As predicted, restoration of miR-342 expression sensitized MCF-7/HER2Δ16 cells to tamoxifen (Figure 5B).

Although miRNAs primarily suppress gene translation, target gene mRNA levels are often suppressed as well [28]. As a global approach to identifying miR-342 target genes we performed gene expression profiling of MCF-7/HER2Δ16 stably transfected with miR-342 compared to vector control cells on a HuGene 1.0 Affymetrix microarray platform. Differentially regulated genes in miR-342-expressing MCF-7/HER2Δ16 cells were defined as those with at least 1.5-fold change (*p *< 0.05) compared to vector expressing cells (Figure 5C). Genes that also changed in vector expressing compared to parental MCF-7/HER2Δ16 cells were excluded. By these criteria miR-342 expressing MCF-7/HER2Δ16 cells showed differential expression of 160 genes, 13 of which are bioinformatically predicted to be direct targets of miR-342 (Table 2). Interestingly, 7 of these 13 genes were upregulated in the miR-342 expressing MCF-7/HER2Δ16 cells (Table 2). The microarray data was validated by performing qRT-PCR of three miR-342 predicted target genes (SEMAD, BMP7, GEMIN4) and TXNIP (Figure 5D) which is not a predicted miR-342 target gene but highly upregulated in miR-342 expressing MCF-7/HER2Δ16 cells. Luciferase expression from the 3' UTR sequences of GEMIN4 and BMP7 was suppressed when coexpressed with miR-342 by 40 and 50%, respectively, suggesting that these genes are direct targets of miR-342 (Figure 5E).

**Table 2:** Target genes significantly modulated by miR-342 expression

Gene Symbol	Gene Title	Fold Change
NR4A2	nuclear receptor subfamily 4, group A, member 2	1.7
MAGED2	melanoma antigen family D, 2	1.7
LASP1	LIM and SH3 protein 1	1.7
UCP2	uncoupling protein 2	1.6
THSD4	thrombospondin, type I, domain containing 4	1.6
PRODH	proline dehydrogenase (oxidase) 1	1.6
PIP4K2C	phosphatidylinositol-5-phosphate 4-kinase, type II, gamma	1.6
MEIS1	meis homeobox 1	-1.7
BMP7	bone morphogenetic protein 7	-1.8
PRR6	proline rich 6	-1.8
GEMIN4	gem associated protein 4	-2.1
GJA1	gap junction protein, alpha 1	-2.2
SEMA3D	semaphorin 3D	-2.3

**Figure 6:**
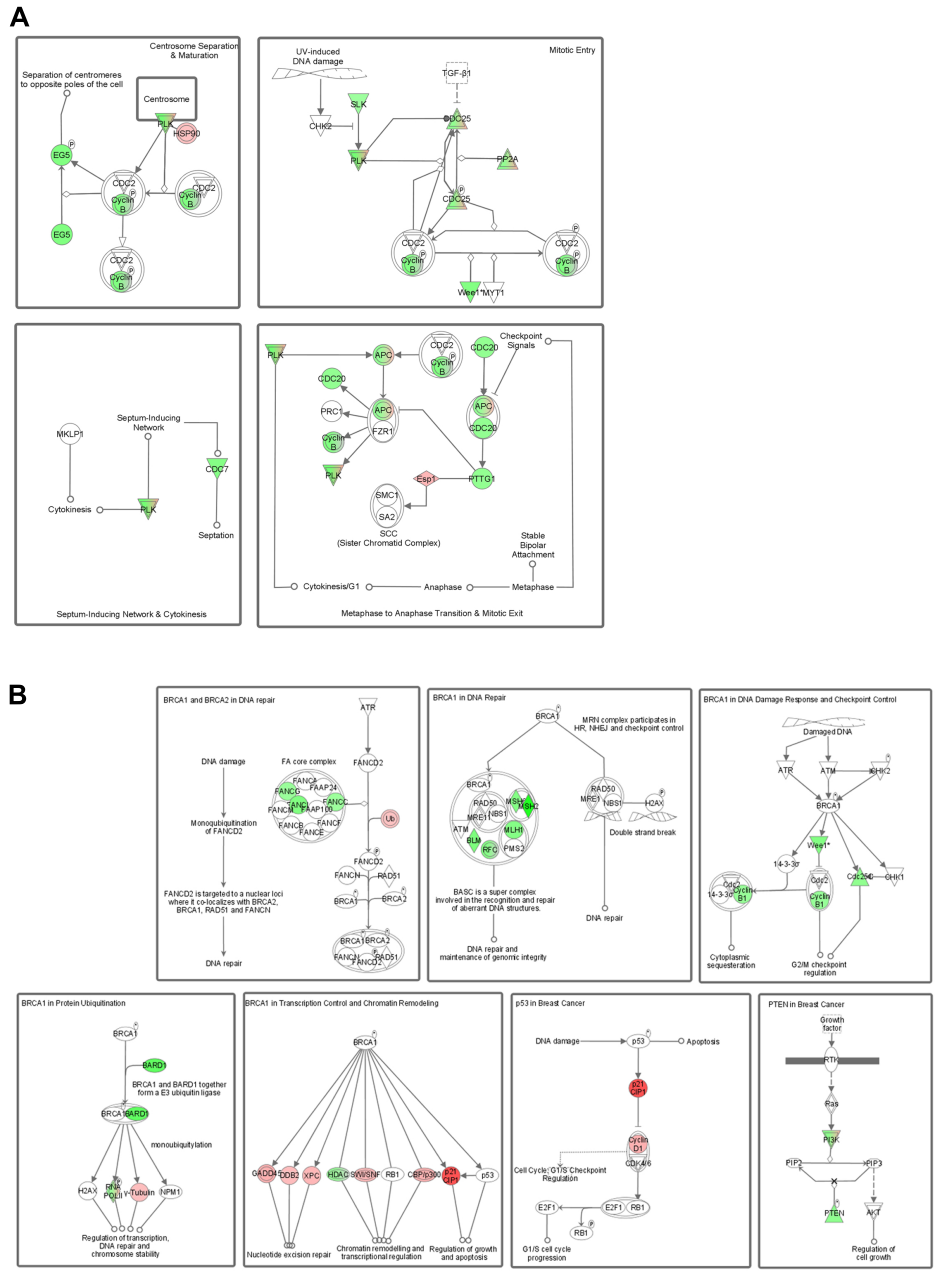
**Ingenuity Pathway Analysis (IPA) of miR-342 regulated genes**. Pathways most significantly enriched with miR-342 regulated genes included (A) Mitotic Roles of Polo-Like Kinase (*p *< 0.0001) and (B) Hereditary Breast Cancer Signaling (*p *< 0.002). Genes significantly upregulated are indicated in red and significantly downregulated genes are indicated in green. Color intensity indicates extent of upregulation or downregulation.

To identify functional pathways potentially modulated by miR-342 expression we performed an Ingenuity Pathway Analysis (version 8.5)(IPA) of the miR-342 regulated gene set. Consistent with a role for miR-342 in tamoxifen induced apoptosis, the "Cell Death" biological function and more specifically "Apoptosis of Breast Cancer Cells" was the biological function most significantly (*p *< 7 × 10^-6^) enriched with miR-342 regulated genes. When canonical cellular pathways were analyzed for miR-342 regulated genes, IPA identified miR-342 genes most significantly enriched in the "Mitotic Roles of Polo-Like Kinase" pathway (*p *< 0.0001)(Figure 6A). Most relevant to breast cancer is the suppression of pathway member cyclin B1 in miR-342 expressing MCF-7/HER2Δ16 cells. Cyclin B1 directly contributes to breast tumor cell proliferation and therapeutic resistance [29]. Furthermore, cyclin B1 expression is an independent negative prognostic factor in the breast cancer clinic [30]. The next breast cancer relevant pathway significantly enriched with miR-342 regulated genes was "Hereditary Breast Cancer Signaling" (*p *< 0.002) which includes multiple genes with obvious connections to breast cancer (Figure 6B). Thus, in addition to a role in apoptosis, our results suggest that miR-342 may also influence multiple stages of the cell cycle through cyclin B1 suppression, multiple BRCA1 activities, p53 cell cycle checkpoint function, and PTEN tumor suppressor activity. All processes that directly impact breast tumorigenesis and tumor cell response to therapeutic intervention.

## Discussion

Endocrine resistance remains an important problem in the breast cancer clinic. Currently there are only a few useful tumor markers to guide management decisions for women with ERα(+) breast tumors. Here we investigated the potential role of miRNAs in the regulation of tamoxifen response with the goal of identifying miRNAs that can be used to predict patient outcome during tamoxifen therapy. We found several miRNAs whose expression was altered in tamoxifen resistant breast tumor cells. We further demonstrate that miR-342-3p (miR-342), an ERα associated miRNA [23], was dramatically suppressed in multiple tamoxifen resistant breast tumor cell lines and in primary breast tumors of patients who failed tamoxifen therapy. Importantly, reintroduction of miR-342 sensitized refractory breast tumor cells to tamoxifen therapy suggesting that miR-342 is an important regulator of tamoxifen response.

Multiple studies have demonstrated that miRNAs are involved in estradiol-dependent breast tumor cell proliferation [31,32]. Accordingly, estradiol regulates expression of many miRNAs [31,32] and an ERα associated miRNA signature has been identified in human breast tumors [23]. In contrast, miRNAs appear to counteract estrogen-dependent cell proliferation and their expression is upregulated by anti-estrogen treatment of breast tumor cells [32]. In this study, we profiled miRNA expression in tamoxifen-sensitive and resistant breast tumor cell lines and we identified several miRNAs differentially regulated in tamoxifen-resistant MCF-7 cells expressing the HER2Δ16 oncogenic isoform of HER2 [14]. In corroboration with recent findings that deregulation of miRNAs contributes to the acquisition of anti-estrogen resistance [11-13], we show that miR-342, which is suppressed in tamoxifen resistant cells, is associated with increased tamoxifen sensitivity of breast tumor cells. Enhanced expression of miR-342 in tamoxifen sensitive breast tumor cells has been reported previously, however, the functional significance of deregulated miR-342 was not investigated by this group [13]. MiR-1308 and miR-1180 were also deregulated by greater than 2-fold in tamoxifen treated MCF-7/HER2Δ16 cells. Studies are in progress to decipher the contribution of these miRNAs to HER2Δ16 mediated endocrine resistance.

It is common for miRNAs located within transcriptional units to be co-expressed with their host gene. Although *in situ *methods for the detection of miRNA expression are not in clinical use, the fact that miR-342 expression is tightly correlated to its host gene EVL in multiple tumor cell models [24,33] including our breast tumor cell lines, provides a unique opportunity to use EVL mRNA expression as a surrogate marker for miR-342 expression in breast cancer patients. Mining of archived microarray data revealed that EVL expression is significantly associated with ERα(+) breast tumors [25-27] which is consistent with recent studies reporting a strong correlation between ERα and miR-342 expression [23]. Significantly, ERα(+) breast tumors that fail tamoxifen therapy with rapid recurrence within 3 years have significantly lower EVL expression levels [34], providing compelling support for the hypothesis that miR-342 suppression promotes tamoxifen resistance. In colorectal cancer cells EVL/miR-342 expression is suppressed through hypermethylation of the *EVL *promoter [24]. We are currently investigating a similar mechanism of miR-342 suppression in tamoxifen resistant breast tumor cells.

A direct gene target of miR-342 remains to be experimentally confirmed and bioinformatics reveals over 1000 potential miR-342 targets. As an approach to the identification of miR-342 targets we examined transcriptome changes in miR-342 overexpressing cells by microarray analysis. We observed significant alteration of 13 genes predicted by bioinformatics to be miR-342 targets. Interestingly, the majority of predicted target genes were up-regulated by miR-342 expression. Although miRNAs commonly suppress target gene mRNA levels, accumulating evidence suggests that miRNAs can target genes for up-regulation by at least two different mechanisms [35]. Although we could not identify an obvious association between the direct targets of miR-342 and tumor cell response to tamoxifen, Ingenuity Pathway Analysis of the entire set of genes significantly altered by miR-342 revealed a highly significant association of miR-342 regulated genes with cell apoptosis. This result is consistent with our observation that ectopic miR-342 expression sensitized tamoxifen resistant cells to tamoxifen-induced apoptosis. Similarly, miR-342 expression in colorectal cancer cells results in tumor cell apoptosis [24]. Nevertheless, activity of miR-342 appears to be functionally different in colorectal and breast tumor cells. Our results indicate that miR-342 expression alone is not sufficient to induce cell death, but miR-342 sensitizes cells to apoptosis associated with estrogen-deprivation and tamoxifen exposure. In this context, the miR-342 indirect target thioredoxin-interacting protein (TXNIP) is the most dramatically upregulated gene in response to miR-342 expression and could potentially mediate miR-342 action. Accordingly, TXNIP expression is induced in tumor cells by various stresses including serum starvation [36], which mimics ERα inactivation in breast tumors, and enhanced TXNIP expression has tumor-suppressor activity [36,37]. Similar to our findings that miR-342 sensitizes breast tumor cells to tamoxifen, TXNIP sensitizes breast tumor cells to paclitaxel [38]. Although these results are compelling, TXNIP is an upregulated indirect target of miR-342 and deciphering the interplay between miR-342 and TXNIP has proved difficult.

Although tamoxifen clearly induces breast tumor cell apoptosis, cytostasis is considered the predominant tumor cell response to tamoxifen therapy. Accordingly, IPA analysis identified miR-342 regulated genes significantly represented in multiple pathways that directly regulate breast tumor cell cycle progression including cyclin B1, p53, and BRCA1. Taken together our results provide a genomic basis to explore the role of miR-342 regulated genes in multiple tamoxifen actions including both apoptosis and cytostasis.

## Conclusions

In summary, we have identified miR-342 as an important mediator of tamoxifen response in both breast tumor cell lines and breast cancer patients. Our results suggest that miR-342 expression and/or expression of the surrogate marker EVL may emerge as important breast tumor markers predicting clinical response to tamoxifen intervention. With the potential of miRNA therapy, restoring miR-342 expression may constitute a novel therapeutic strategy to sensitize endocrine refractory breast tumors.

## List of Abbreviations

CS-FBS MEM: charcoal stripped fetal bovine serum in phenol red free MEM; E2: 17-β-estradiol; ERα: estrogen receptor alpha; EVL: Ena/Vasp-like; FFPE: formalin fixed paraffin embedded; HER2Δ16: oncogenic splice isoform of HER2; IPA: ingenuity pathway analysis; ISH: *in situ *hybridization; miRNA: micro RNA; miRNAs: micro RNAs; miR-342: miR-342-3p; MTT: 3-(4,5-dimethylthiazol-2-yl])-2,5-diphenyltetrazolium bromide; PI: propidium iodine; qRT-PCR: quantitative reverse transcriptase polymerase chain reaction; TAM: 4-hydroxytamoxifen; TXNIP: thioredoxin-interacting protein; UTR: untranslated region

## Competing interests

The authors declare that they have no competing interests.

## Authors' contributions

DMC performed all miR-342 and apoptosis analyses as well as prepared the manuscript draft. PMD performed all miR-342 and cell growth analyses required for the revised manuscript. NSS and SME performed miR-342 ISH experiments. JKR and ADT evaluated and monitored ISH experiments. FEJ conceived of the study, participated in data analysis at each stage, and finalized preparation of the manuscript. All authors read and approved the final manuscript.

## Supplementary Material

Additional file 1 **Levels of miR-342-3p and miR-342-5p expression in breast tumor cell lines**. File shows qRT-PCR data comparing levels of miR-342-3p and miR-342-5p expression in experimental cell lines. Total RNA was isolated from each cell line and miR-342-3p or miR-342-5p expression levels relative to GAPDH were analyzed by qRT-PCR. Data is represented as mean +/- SE of three independent RNA extractions. Click here for file

Additional file 2 **Scoring of miR-342 expression by ISH in primary breast tumors and breast tumor cell lines**. File contains controls for ISH showing different levels of miR-342 expression and ISH scoring. (A-D) ISH for miR-342 expression was performed and scored on each primary breast tumor as described in the materials and methods. The samples are scored as (A) 0, (B) 1, (C) 2, and (D) 3. (E) Total RNA was isolated from each cell line and miR-342 expression levels analyzed by qRT-PCR were normalized to RNU44. Data relative to untreated MCF-7/HER2Δ16 is represented as mean +/- SE of three independent RNA extractions. (F) Mir-342 ISH was performed on each formalin fixed and paraffin embedded cell line. Indicated fold changes in miR-342 expression were obtained from the qRT-PCR from E. Click here for file

Additional file 3 **Levels of miR-342-3p and miR-342-5p expression in MCF-7/HER2Δ16 cell lines**. File shows qRT-PCR data comparing levels of miR-342-3p and miR-342-5p expression in MCF-7/HER2Δ16 stable cell lines. Total RNA was isolated from each cell line and miR-342-3p or miR-342-5p expression levels relative to RNU44 were analyzed by qRT-PCR. Data is represented as mean +/- SE of three independent RNA extractions. Levels of miR-342-5p expression were at the lower limits of qRT-PCR sensitivity. Click here for file
