# Supplementary figures and images for: Downregulation of miR-342 is associated with tamoxifen resistant breast tumors
Source: Mol Cancer. 2010 Dec 20;9:317. doi: 10.1186/1476-4598-9-317 (PMC3024251; doi:10.1186/1476-4598-9-317)

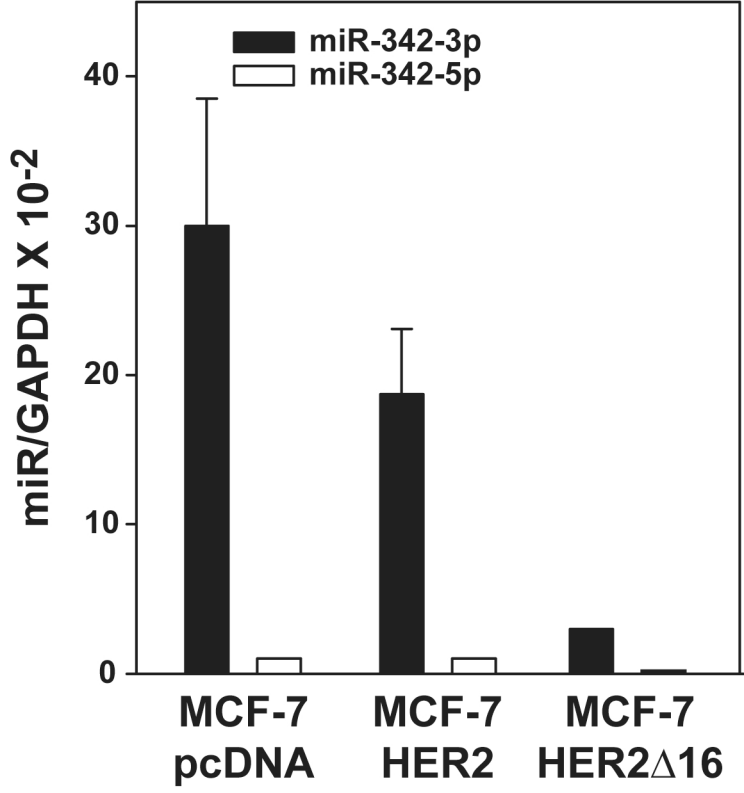

Supplement: Additional file 1 — Levels of miR-342-3p and miR-342-5p expression in breast tumor cell lines. File shows qRT-PCR data comparing levels of miR-342-3p and miR-342-5p expression in experimental cell lines. Total RNA was isolated from each cell line and miR-342-3p or miR-342-5p expression levels relative to GAPDH were analyzed by qRT-PCR. Data is represented as mean +/- SE of three independent RNA extractions. [file 1476-4598-9-317-S1.PDF]

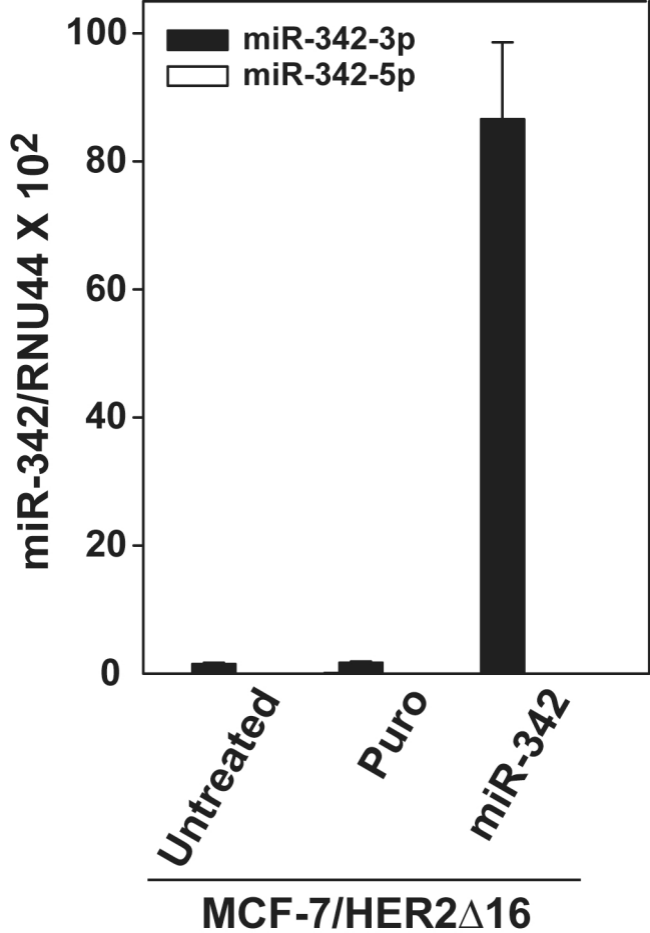

Supplement: Additional file 2 — Scoring of miR-342 expression by ISH in primary breast tumors and breast tumor cell lines. File contains controls for ISH showing different levels of miR-342 expression and ISH scoring. (A-D) ISH for miR-342 expression was performed and scored on each primary breast tumor as described in the materials and methods. The samples are scored as (A) 0, (B) 1, (C) 2, and (D) 3. (E) Total RNA was isolated from each cell line and miR-342 expression levels analyzed by qRT-PCR were normalized to RNU44. Data relative to untreated MCF-7/HER2Δ16 is represented as mean +/- SE of three independent RNA extractions. (F) Mir-342 ISH was performed on each formalin fixed and paraffin embedded cell line. Indicated fold changes in miR-342 expression were obtained from the qRT-PCR from E. [file 1476-4598-9-317-S2.PDF]

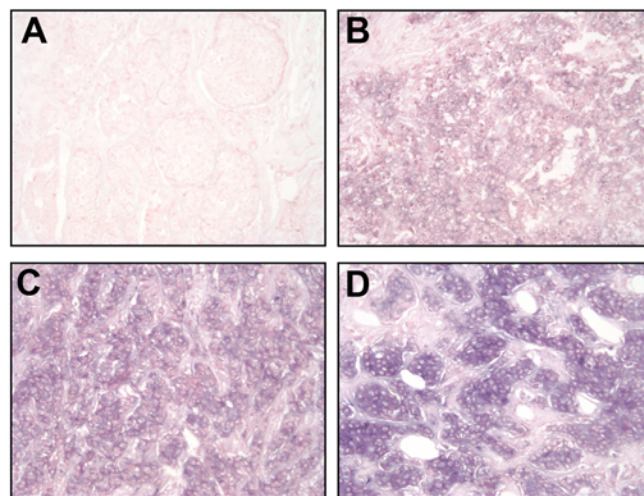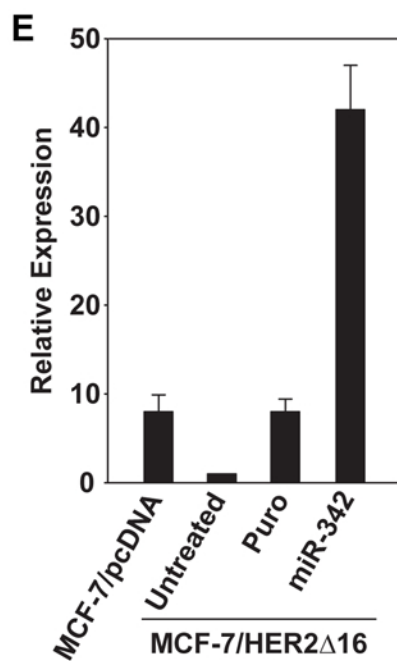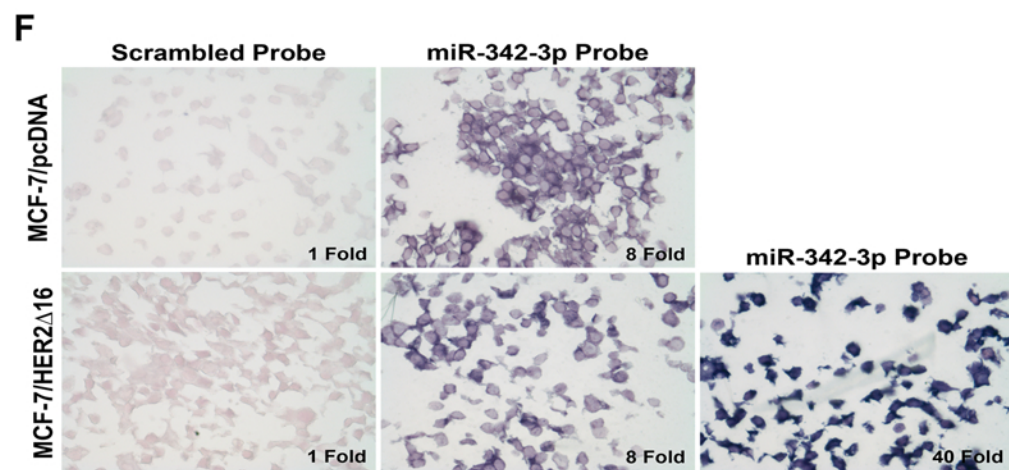

Supplement: Additional file 3 — Levels of miR-342-3p and miR-342-5p expression in MCF-7/HER2Δ16 cell lines. File shows qRT-PCR data comparing levels of miR-342-3p and miR-342-5p expression in MCF-7/HER2Δ16 stable cell lines. Total RNA was isolated from each cell line and miR-342-3p or miR-342-5p expression levels relative to RNU44 were analyzed by qRT-PCR. Data is represented as mean +/- SE of three independent RNA extractions. Levels of miR-342-5p expression were at the lower limits of qRT-PCR sensitivity. [file 1476-4598-9-317-S3.PDF]
